# Women’s Attitudes Toward Invasive and Noninvasive Testing When Facing a High Risk of Fetal Down Syndrome

**DOI:** 10.1001/jamanetworkopen.2019.1062

**Published:** 2019-03-29

**Authors:** Valerie Seror, Olivier L’Haridon, Laurence Bussières, Valérie Malan, Nicolas Fries, Michel Vekemans, Laurent J. Salomon, Yves Ville

**Affiliations:** 1 Aix Marseille Univ, IRD, AP-HM, SSA, VITROME, Marseille, France; 2 IHU Méditerranée Infection, Marseille, France; 3 CREM, Université de Rennes 1, Rennes, France; 4 Clinical Unit Research/Clinic Investigation Center, Paris Descartes, Hôpital Necker-Enfants Malades, Assistance Publique-Hôpitaux de Paris, Paris, France; 5 Department of Obstetrics and Gynecology, Hôpital Necker-Enfants Malades, Assistance Publique-Hôpitaux de Paris, Paris, France; 6 Department of Histology-Embryology and Cytogenetics, Hôpital Necker-Enfants Malades, Assistance Publique-Hôpitaux de Paris, Paris, France; 7 INSERM U1163, Hôpital Necker-Enfants Malades, Paris, France; 8 Paris Descartes University, Sorbonne Paris Cité, Institut Imagine, Paris, France; 9 Department of Obstetrics and Gynecology, CHU de Montpellier, Montpellier, France; 10 Collège Français d'Echographie Fœtale, Chateaubriand, France; 11 Groupe de Recherche en Obstétrique et Gynécologie, Paris, France; 12 Collège Français d'Echographie Fœtale, Chateaubriand, France

## Abstract

**Importance:**

Noninvasive prenatal testing (NIPT) using cell-free DNA in maternal blood is increasingly common compared with invasive testing (IT) in routine antenatal detection of Down syndrome (DS).

**Objective:**

To assess attitudes and decision making in pregnant women facing a risk of fetal DS greater than 1 in 250 as established by combined first trimester screening at 11 to 14 weeks of gestation.

**Design, Setting, and Participants:**

Survey study in which data were collected from pregnant women at high risk of fetal DS participating in a randomized clinical trial. Data were collected from April 8, 2014, to April 7, 2016, in 57 prenatal diagnosis centers in France. Data were analyzed in 2018.

**Interventions:**

Data on attitudes were collected prior to offering randomization between NIPT and IT, whereas data on decision making and test results were collected as part of the clinical trial.

**Main Outcome and Measures:**

The primary outcome related to attitudes. A hierarchical cluster analysis was conducted to identify clusters with contrasting attitudes. Logistic regression analyses were used to identify factors associated with attitudes.

**Results:**

All 2436 consecutive women to whom the study was proposed (mean [SD] age, 36.3 [5.0] years) answered the questionnaire: 515 (21.1%) expressed preference toward IT with complete karyotyping, whereas 1843 (75.7%) favored NIPT with almost certain but limited information. Hierarchical cluster analysis yielded 4 different clusters that mainly differed in attitudes toward risk taking and extent of information seeking. Factors likely associated with attitudes driven by risk aversion were mostly age and religious beliefs (adjusted odds ratio [aOR], 1.03; 95% CI, 1.00-1.05; *P* = .03 and aOR, 1.62; 95% CI, 1.29-2.04; *P* < .001, respectively), whereas higher nuchal translucency measurements by ultrasonography were associated with attitudes driven by ambiguity aversion (aOR, 1.67; 95% CI, 1.27-2.20; *P* < .001). For attitudes involving both risk and ambiguity aversion at different extents, lower education was associated with highly valuing all possibilities of getting information on pregnancy, whereas higher education was associated with highly valuing information on fetal DS as a primary concern (aOR, 0.54; 95% CI, 0.44-0.67; *P* < .001 and aOR, 1.44; 95% CI, 1.20-1.74; *P* < .001, respectively). In all, decision making was in line with attitudes.

**Conclusions and Relevance:**

Aversion to risk of fetal loss related to IT and aversion to ambiguity generated by incomplete information from NIPT played a major role in shaping attitudes and decision making. Informed decision making should require pregnant women at high risk of DS to receive extensive information on targeted abnormalities by both tests.

## Key Points

**Question:**

What are the attitudes and decision making concerning invasive and noninvasive prenatal testing in women at high risk of fetal Down syndrome?

**Findings:**

In a survey study of 2436 pregnant women in France participating in a randomized clinical trial, 4 clusters were identified with different attitudes toward risk taking and extent of information seeking. Decision making was in line with attitudes, and clinical and socioeconomic factors were likely associated with the attitudes identified.

**Meaning:**

Aversion to ambiguity generated by incomplete information from noninvasive testing as well as aversion to risk of pregnancy loss due to invasive testing played a major role in shaping attitudes and decision making; therefore, pregnant women should receive extensive information on targeted abnormalities by both tests to aid informed decision making.

## Teaser

This survey study examines the attitudes of pregnant women in France at high risk of fetal Down syndrome toward invasive and noninvasive prenatal testing.

## Introduction

Prenatal screening for Down syndrome (DS) is widely performed at 11 to 14 weeks of gestation by using a combination of maternal age, measurements of fetal nuchal translucency by ultrasonography, and maternal serum concentrations of free human chorionic gonadotropin and plasma protein A.^1^ Various cutoffs of risk have been chosen to offer invasive testing (IT) as a second step, usually between 1 in 100 and 1 in 300.^1^ More recently, noninvasive prenatal testing (NIPT) using cell-free DNA circulating in maternal plasma has emerged as an intermediate step within a conditional screening algorithm, as the positive predictive value of combined screening is around 1 in 16 for DS.^2,3,4,5,6,7^ In contrast with IT, which involves a risk of miscarriage but provides full information on all possible chromosomal abnormalities, NIPT carries no risk of miscarriage but only provides information on the targeted abnormalities and only in terms of very high likelihood of their presence or absence. Indeed, the accuracy of cell-free-DNA to detect DS is around 99% with a 0.1% false-positive rate, and IT is carried out to confirm a positive result.^8^ As a result, NIPT therefore involves both a risky, although almost certain, component about targeted abnormalities together with an uncertain component about nontargeted abnormalities. In addition to the well-known attractiveness of certainty in decision making (also known as the certainty effect^9,10^), presenting information involving both risky and uncertain components is known to trigger ambiguity aversion^11,12^ as a result of a preference for known risks (probabilities) over unknown risks.^13^ Decision making between IT and NIPT could therefore be driven by aversion toward ambiguous information as much as by IT-related pregnancy loss risk aversion.

The present study aimed to explore attitudes of pregnant women who are about to make their decision following positive combined screening for DS. Considering the question addressed about the possible role of ambiguity aversion in addition to risk aversion in shaping preferences and decision making, this study involved assessing attitudes, factors likely associated with attitudes, and consistency between attitudes and actual decision making in high-risk women invited to participate in a multicenter randomized trial of IT vs NIPT.^14^

## Methods

Data on pregnant women’s attitudes were collected within the framework of a randomized clinical trial comparing risk and effectiveness of NIPT and IT at a national level, involving 57 prenatal diagnosis centers (ClinicalTrials.gov identifier NCT02127515). Women with a fetal DS gestation-specific risk of 1 in 250 or greater following combined screening were invited to enroll in the clinical trial. They received one-on-one extensive information and counseling on the significance of this result as well as on advantages and risks attached to both IT and NIPT options.^14^ More specifically, the information provided to all participants as to the risks and benefits of each group of the study included issues relating to culture failure and mosaicism with IT as well as the chance of false-negative results with NIPT. The extent of counselling was intended to minimize the role of the heterogeneity of women’s access to relevant information prior to prenatal screening. Pregnant women were then asked to fill out a self-administered electronic questionnaire on site (eAppendix in the Supplement) exploring their attitude toward IT and NIPT in this high-risk situation. They were then asked to participate in the randomized clinical trial comparing NIPT and IT. Participants provided written informed consent and the study received ethics committee approval as part of the multicenter randomized trial. This study followed the American Association for Public Opinion Research (AAPOR) reporting guideline.

Exclusion criteria were multiple pregnancies and nuchal translucency measurements over 3 mm, as this could be a marker of other submicroscopic chromosomal disorders that would not be detectable by NIPT and only by IT. Another exclusion criterion was the absence of medical coverage by the French national health insurance system. Women who declined to participate in the trial, whether they refused prior to randomization between IT vs NIPT or their randomized assignment to NIPT, still had the possibility of undergoing IT without extra charges because these women remained eligible for full coverage by the French national health insurance system. On the contrary, women who refused their randomized assignment to IT and then decided to undergo NIPT had to pay for it. As a result, opting for IT after negative NIPT led to pregnancy risk taking without financial cost, whereas declining IT to opt for NIPT did not involve pregnancy risk taking but was financially costly.

### Questionnaire

In addition to sociodemographic characteristics, the questionnaire included 23 questions on decision making about DS screening, understanding of combined screening test results, and attitudes toward IT-related fetal loss as compared with the birth of a child with DS. Specifically, the questionnaire addressed attitudes about the trade-off between risk taking and extent of information seeking, preferences between carrying out IT and NIPT, preference-based measurements using visual analog scales (VAS; from 0-10 with higher or lower scores indicating stronger preference for IT or NIPT, respectively), and decisional attitudes. Risk of fetal loss related to IT was rated to be around 1% as it is mentioned on French informed consent forms. Lastly, clinical data were available from the trial, including nuchal translucency measurements, calculated risk of DS from combined screening, gravidity, parity, smoking status, and IT uptake and diagnosis of chromosomal abnormalities in previous pregnancies. Miscarriages in previous pregnancies were also identified.

### Statistical Analysis

Women’s attitudes were assessed using a hierarchical cluster analysis^15^ aiming to allocate individuals to groups as suggested by the recorded data; these groups were therefore not defined a priori. Using the Euclidean metric to compute distances between individuals and to cluster them,^16^ the agglomerative hierarchical procedure first involved each observation to be a cluster by itself. Then, the 2 closest clusters were merged to form a new one, and this procedure was repeated until only 1 cluster was left.^17^ The optimum number of clusters was determined using a 2-fold criterion: minimization of the interindividual distances within a cluster (within-group homogeneity) and maximization of the distances between the centers of gravity of clusters (between-cluster heterogeneity). Individuals in a given cluster tended to be similar for the variables selected for analysis, while individuals with contrasting characteristics were allocated to different clusters. Typical patterns of attitudes were thus identified as a result of the hierarchical cluster analysis. Each cluster was characterized by the variables that contributed significantly to its formation, using *P* values calculated by multiscale bootstrap resampling methods. Because of collinearity issues between the variables relating to women’s preferences and associated VAS scores, VAS scores were used as an illustrative variable that was not involved in the calculations but was used to illustrate statistical results. The hierarchical cluster analysis was carried out using the SPAD software package (Coheris).

Statistical analysis also involved logistic regressions to assess the relative effects of various factors on women’s attitudes. All variables associated with a *P* value less than .10 in the univariate analyses were taken to be eligible for the multivariable analysis. Using a backward selection procedure, only variables remaining independently and significantly associated with the outcome with a 2-tailed *P* value less than .05 were kept in the final model.

## Results

The present study was proposed to 2592 consecutive pregnant women at high risk for fetal DS from April 8, 2014, to April 7, 2016. Among these women, 33 declined to participate in the trial and refused to fill out the questionnaire, whereas 123 participated in the trial but did not fill out the questionnaire. As a result, 2436 questionnaires of consecutive pregnant women at high risk for DS were studied (Figure).

**Figure.**
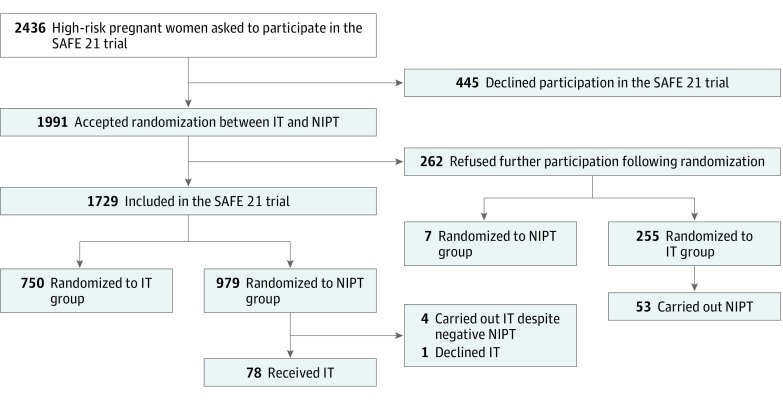
Participant Flowchart IT indicates invasive testing; NIPT, noninvasive prenatal testing.

Clinical and sociodemographic characteristics of the women together with their understanding of their own risk of fetal DS are presented in Table 1. Overall, mean (SD) maternal age was 36.3 (5.0) years, 12.6% had IT in a previous pregnancy, and 4.2% experienced fetal chromosomal abnormalities in a previous pregnancy. Fetal risk of DS ranged from 0.004 to 0.20 (mean, 0.01). In addition, two-thirds of the women reached at least upper secondary educational level, and about a third reported religion to be very or fairly important to them. Self-assessment of their level of risk for DS was consistent with the calculated risk from combined screening (*P* < .001): a 0.0203 average calculated risk was observed in the pregnant women who considered their risk as very high, whereas a 0.0101 and 0.0075 average calculated risk was associated to perceived risk as high or rather low, respectively. However, women with an educational level below secondary school graduation were more likely than others to consider their risk to be rather low (36.1% vs 21.2%; *P* < .001). In addition, women unable to appraise their level of risk were more likely to be at lower risk (mean risk at 0.0088 vs 0.0110; *P* = .02).

**Table 1.** Clinical and Sociodemographic Characteristics, Understanding of the Results of DS Combined Screening Test, and Attitudes Toward IT and NIPT of 2436 Pregnant Women at High Risk for Fetal DS

Characteristic	No (%)
Age, mean (SD), y	36.3 (5.0)
Nuchal translucency measurement, mean (SD), multiple of the median	0.6901 (0.5034)
DS risk: combined test results, mean (SD), multiple of the median	0.0108 (0.0124)
No prior pregnancy	604 (24.4)
Nulliparous	799 (32.8)
Invasive testing in previous pregnancies	306 (12.6)
Diagnosis of fetal chromosomal abnormalities in previous pregnancies	103 (4.2)
Current smoker	441 (18.1)
Time of inclusion with respect to first inclusion, mean (SD), d	382.11 (182.433)
Educational level	
No education or primary education	146 (6.0)
Lower secondary education	449 (18.4)
Secondary education	359 (14.7)
Upper secondary education	435 (17.9)
Postsecondary and tertiary education	1003 (41.2)
Missing	44 (1.8)
Religion	
Very important	289 (11.9)
Fairly important	426 (17.5)
Not very important	615 (25.2)
Not important at all	1039 (42.6)
Missing	67 (2.7)
Women’s understanding of combined test	
Rather low risk	611 (25.1)
High risk	1256 (51.6)
Very high risk	347 (14.2)
Other: comments on screening and DS risk	181 (7.4)
Missing	41 (1.7)
Decision-making about biochemical screening	
Decided to carry it out before the option was proposed	1030 (42.3)
Did not think about it before the option was proposed	1000 (41.0)
Did not know about it before the option was proposed	392 (16.1)
No opinion	14 (0.6)
While awaiting the combined test results, anticipated decision about invasive testing?	
Yes	726 (29.8)
No	1680 (69.0)
No opinion	30 (1.2)
Worst pregnancy outcome, in the women’s opinion	
Birth of a child with DS	1371 (56.3)
Miscarriage due to invasive testing	933 (38.3)
No opinion	132 (5.4)
What is best?	
To take the risk of procedure-related fetal loss (occurring in approximately 1 woman in 100) and obtain certain information on all possible chromosomal abnormalities	976 (40.1)
Not to take the risk of procedure-related fetal loss and get almost certain information (99% reliability) on DS only	1388 (57.0)
No opinion	72 (3.0)
Women’s preferences between IT and NIPT	
Neither IT nor NIPT	29 (1.2)
NIPT	1843 (75.7)
IT	515 (21.1)
No opinion	49 (2.7)
Visual analog scale score, mean (SD)^a^	3.09 (3.25)
Decisional preferences after having been informed about the trial	
To decline both IT and NIPT	25 (1.0)
To carry out NIPT if offered and to decline IT otherwise	357 (14.7)
To carry out NIPT provided also carrying out IT	1758 (72.2)
To carry out IT and to decline NIPT	218 (8.9)
No opinion	78 (3.2)
It is best to take the risk of procedure-related fetal loss rather than to be worried until the end of the pregnancy	
Strongly agree	872 (35.8)
Rather agree	1019 (41.8)
Rather disagree	346 (14.2)
Strongly disagree	182 (7.5)
No opinion	17 (0.7)
Having carried out biochemical screening should lead women to undergo invasive testing if it is advised by their health care professional	
Strongly agree	851 (34.9)
Rather agree	986 (40.5)
Rather disagree	372 (15.3)
Strongly disagree	204 (8.4)
No opinion	23 (0.9)
Invasive testing should be declined when termination of pregnancy is not an option in the case of DS	
Strongly agree	713 (29.3)
Rather agree	681 (28.0)
Rather disagree	552 (22.7)
Strongly disagree	466 (19.1)
No opinion	24 (1.0)
Invasive testing should be carried out without considering the decisions that might be required following its results: everything in its own time	
Strongly agree	607 (24.9)
Rather agree	778 (31.9)
Rather disagree	641 (26.3)
Strongly disagree	389 (16.0)
No opinion	21 (0.9)

Abbreviations: DS, Down syndrome; IT, invasive testing; NIPT, noninvasive prenatal testing.

^a^ Visual analog scale from 0 to 10, with lower values indicating stronger preference for NIPT.

### Attitudes Toward NIPT and IT

Using data relating to pregnant women’s attitudes (Table 1), the hierarchical cluster analysis yielded 4 different clusters. The variables characterizing each one of these clusters are presented in Table 2. The first cluster (10.3% [250 of 2436 respondents]) consisted of women who highly valued the possibility of getting all possible information on chromosomal abnormalities without any delay. These women reported more frequently than the others that they had anticipated the possibility of having to decide about IT in case of a high risk of DS (36.8% vs 28.8%; difference, 8.0%; 95% CI, 17.4%-14.6%; *P* = .006). Most of them considered the risk of IT-related fetal loss to be acceptable while providing access to all possible information on fetal chromosomal abnormalities (85.6% vs 34.9%; difference, 50.7%; 95% CI, 45.2%-55.2%; *P* < .001) and preventing further anxiety (52.0% vs 33.9%; difference, 18.1%; 95% CI, 11.4%-24.7%; *P* < .001). Women in this cluster expressed a clear-cut preference for IT (79.6% vs 14.5%; difference, 65.1%; 95% CI, 59.3%-70.1%; *P* < .001) and considered declining NIPT if offered (87.3% vs 0%; difference, 87.3%; 95% CI, 82.3%-91.0%; *P* < .001).

**Table 2.** Variables Contributing to Distinguishing Clusters of 2436 Pregnant Women at High Risk for Fetal DS^a^

Variable	Total, No. (%)	Cluster 1 (n = 250)		Cluster 2 (n = 485)		Cluster 3 (n = 1324)		Cluster 4 (n = 377)	
		No. (%)	*P* Value	No. (%)	*P* Value	No. (%)	*P* Value	No. (%)	*P* Value
Decided to carry out biochemical screening before it was proposed	1030 (42.3)	114 (45.6)	.16	174 (35.9)	.001	604 (45.6)	<.001	138 (36.6)	.008
While awaiting the combined test results, anticipated the decision about invasive testing	726 (29.8)	92 (36.8)	.006	149 (30.7)	.33	384 (29.0)	.20	101 (26.8)	.08
Decisional preferences									
To carry out IT and to decline NIPT	218 (8.9)	218 (87.3)	<.001	0	<.001	0	<.001	0	<.001
To carry out NIPT provided also carrying out IT	1758 (72.2)	0	<.001	461 (95.1)	<.001	1297 (98.0)	<.001	0	<.001
To carry out NIPT and to decline IT	357 (14.7)	0	<.001	10 (2.1)	<.001	0	<.001	347 (92.0)	<.001
The birth of a child with DS is a worse pregnancy outcome than IT-related miscarriage	1371 (56.3)	177 (70.8)	<.001	310 (63.9)	<.001	779 (58.8)	.003	105 (27.9)	<.001
Strongly agreed that it is best to take the risk of IT-related fetal loss rather than to be worried until the end of the pregnancy	872 (35.8)	130 (52.0)	<.001	260 (53.6)	<.001	443 (33.5)	.004	39 (10.3)	<.001
Strongly agreed that having carried out biochemical screening should lead women to undergo IT if advised	851 (34.9)	101 (40.2)	.04	283 (58.4)	<.001	406 (30.7)	<.001	61 (16.2)	<.001
Strongly agreed that IT should be declined when termination of pregnancy is not an option in the case of DS	713 (29.3)	80 (32.0)	.19	130 (26.8)	.10	370 (27.9)	.07	133 (35.3)	.004
Strongly agreed that IT should be carried out without considering the decisions that might be required following its results	607 (24.9)	65 (26.0)	.04	485 (100)	<.001	0	<.001	57 (15.1)	<.001
It is best to take the risk of IT-related fetal loss and get certain information on all possible chromosomal abnormalities	976 (40.1)	214 (85.6)	<.001	243 (50.1)	<.001	475 (35.9)	<.001	44 (11.7)	<.001
Preference for IT over NIPT^b^	515 (21.1)	199 (79.6)	<.001	91 (18.8)	.08	204 (15.4)	<.001	21 (5.6)	<.001
VAS score, mean (SD)	3.09 (3.25)	7.56 (2.81)		3.15 (3.11)		2.80 (2.81)		1.09 (2.33)	
VAS scores ≥5	816 (33.5)	223 (89.2)	<.001	184 (37.9)	.02	374 (28.2)	<.001	35 (9.3)	<.001

Abbreviations: DS, down syndrome; IT, invasive testing; NIPT, noninvasive prenatal testing; VAS, visual analog scale.

^a^ Cluster 1 was characterized by participants who wanted all possible information as soon as possible (250 women [10.3%]); cluster 2, participants who wanted all possibilities of getting information (485 women [19.9%]); cluster 3, participants who were primarily concerned with information on DS (1324 women [54.3%]); and cluster 4, participants characterized by aversion to risk of fetal loss (377 women [15.5%]).

^b^ The VAS scores are used as an illustrative variable.

The second cluster (19.91% [485 respondents]) included women who valued all possibilities of getting information on their pregnancy. They frequently stated, as in the previous cluster, that the birth of a baby with DS was a worse pregnancy outcome than IT-related fetal loss (63.9% vs 54.4%; difference, 9.5%; 95% CI, 4.5%-14.3%; *P* < .001) and that getting all possible information on chromosomal abnormalities was worth the risk of fetal loss (50.1% vs 37.6%; difference, 12.5%; 95% CI, 7.5%-17.6%; *P* < .001). While women in this cluster also deemed that having undergone DS screening should prompt women to carry out IT if offered (58.4% vs 29.1%; difference, 29.3%; 95% CI, 24.2%-34.1%; *P* < .001), they did not express clear-cut preference between IT and NIPT (18.8% vs 21.7%; difference, 2.9%; 95% CI, −1.3% to 6.8%; *P* = .08) and they accordingly viewed carrying out both NIPT and then IT as their preferred option (95.1% vs 66.5%; difference, 28.6%; 95% CI, 25.4%-31.3%; *P* < .001).

The third cluster (54.4% [1324 respondents]) included women who primarily valued getting information on fetal DS. Women in this cluster had decided more frequently than the others to undergo biochemical screening before it was even proposed (45.6% vs 38.3%; difference, 7.3%; 95% CI, 3.3%-11.3%; *P* < .001). Like in previous clusters, most women considered that the birth of a baby with DS was a worse pregnancy outcome than IT-related fetal loss (58.8% vs 53.2%; difference, 5.6%; 95% CI, 1.6%-9.6%; *P* = .003). However, all of them deemed that IT should not be undergone without considering the decisional implications (100% vs 45.4%; difference, 54.6%; 95% CI, 51.6%-57.5%; *P* < .001) and they also frequently stated that getting complete information on all possible chromosomal abnormalities was not worth the risk for the pregnancy (64.1% vs 54.9%; difference, 9.2%; 95% CI, 5.2%-13.1%; *P* < .001). While they expressed preference toward NIPT (84.6% vs 72.0%; difference, 12.6%; 95% CI, 9.2%-15.9%; *P* < .001), they did not reject the IT option at the time when they were offered to participate in the trial because nearly all considered carrying out IT following a negative NIPT (98.0% vs 41.5%; difference, 56.5%; 95% CI, 53.4%-59.5%; *P* < .001).

The fourth cluster (15.5% [377 respondents]) consisted of women whose attitudes appeared to be driven by aversion to the risk of IT-related fetal loss (72.1% vs 38.5%; difference, 33.6%; 95% CI, 28.3%-38.5%; *P* < .001). Accordingly, most of them would choose not to take any risk with the pregnancy and get information on DS only (88.3% vs 54.7%; difference, 33.6%; 95% CI, 28.3%-38.5%; *P* < .001). Women in this cluster expressed a clear-cut preference for NIPT (94.4% vs 76.0%; difference, 18.4%; 95% CI, 15.0%-21.2%; *P* < .001) and approximately one-third of them deemed that IT should be declined when termination of the pregnancy is not an option (35.3% vs 28.2%; difference, 7.1%; 95% CI, 1.9%-12.6%; *P* = .004).

Perceived values attached to IT and NIPT as rated with a VAS showed the highest scores for IT were found in cluster 1 (89.2% of VAS scores greater than 5 vs 27.1% in the other clusters; difference, 62.1%; 95% CI, 57.0%-66.0%; *P* < .001) while the lowest scores were found in cluster 4 (9.3% vs 37.9%; difference, 28.6%; 95% CI, 13.0%-19.5%; *P* < .001). Otherwise, women in cluster 2 had higher VAS scores than women in cluster 3, which was also consistent with more frequent ratings greater than 5 in cluster 2 (37.9% vs 28.2%; difference, 9.7%; 95% CI, 4.7%-14.8%; *P* = .02).

### Factors Associated With Attitudes

The factors associated with women’s attitudes toward NIPT and IT are presented in Table 3. Women with higher nuchal translucency measurements by ultrasonography were more likely to be included in cluster 1 (adjusted odds ratio [aOR], 1.67; 95% CI, 1.27-2.20; *P* < .001), whereas cluster 2 was more likely to include women with lower educational levels (aOR, 0.54; 95% CI, 0.44-0.67; *P* < .001). Women in cluster 3 were characterized by higher educational levels (aOR, 1.44; 95% CI, 1.20-1.74; *P* < .001) and lower importance given to religion (aOR, 0.68; 95% CI, 0.57-0.82; *P* < .001). Lastly, older women (aOR, 1.03; 95% CI, 1.00-1.05; *P* = .03), those who described their own risk of DS as rather low (aOR, 1.34; 95% CI, 1.05-1.71; *P* = .02), and those with very or fairly strong religious beliefs (aOR, 1.62; 95% CI, 1.29-2.04; *P* < .001) were more likely to be included in cluster 4. Allocations to cluster 1 decreased slightly over time throughout the trial (aOR, 0.99; 95% CI, 0.99-0.99; *P* < .001), whereas allocations increased slightly over time in cluster 3 (aOR, 1.01; 95% CI, 1.00-1.01; *P* = .009).

**Table 3.** Factors Associated With Attitudes to NIPT and IT Among Women at High Risk for Fetal DS^a^

Factor	Cluster 1 (n = 250)				Cluster 2 (n = 485)				Cluster 3 (n = 1324)				Cluster 4 (n = 377)			
	No. (%)	*P* Value^b^	aOR (95% CI)	*P* Value	No. (%)	P Value^b^	aOR (95% CI)	*P* Value	% or Mean (SD)	*P* Value^b^	aOR (95% CI)	*P* Value	% Mean (SD)	*P* Value^b^	aOR (95% CI)	*P* Value
Nuchal translucency measurement, mean (SD), multiple of the median	0.8040 (0.4543)	<.001	1.67 (1.27-2.20)	<.001	0.6866 (0.4945)	.87			0.6798 (0.5101)	.27			0.6552 (0.5136)	.14		
Calculated risk from combined test		.06				.98				.84				.06		
≥1 in 50	39 (15.6)	.02			55 (11.3)	.85			147 (11.1)	.90			29 (7.7)	.02		
1 in 100 to 1 in 50 [reference]	41 (16.4)				83 (17.1)				233 (17.6)				62 (16.5)			
<1 in 100	170 (68.0)	.98			347 (71.6)	.98			944 (71.3)	.56			286 (75.9)	.43		
Women’s age, mean (SD), y	35.7 (5.3)	.04			36.7 (5.0)	.07			36.1 (4.9)	.07			36.8 (5.1)	.03	1.03 (1.00-1.05)	.03
IT in previous pregnancies	30 (12.0)	.78			66 (13.6)	.44			167 (12.6)	.93			43 (11.4)	.46		
Diagnosed fetal chromosomal abnormalities in previous pregnancies	12 (4.8)	.64			24 (5.0)	.38			53 (4.0)	.55			14 (3.7)	.59		
Previous pregnancy vs none	61 (24.4)	.06			74 (15.3)	.005	0.74 (0.56-0.98)	.03	283 (21.4)	.04			66 (17.5)	.21		
Nulliparous vs multiparous	82 (32.8)	.32			123 (25.4)	.01			411 (31.0)	.24			116 (30.8)	.74		
>1 Fetal loss in previous pregnancies	33 (13.2)	.04	0.69 (0.47-1.02)	.06	99 (20.4)	.11			225 (17.0)	.19			80 (21.2)	.07		
Women’s own understanding of their DS risk as rather low	50 (20.0)	.05			138 (28.5)	.06			311 (23.5)	.048			112 (29.7)	.03	1.34 (1.05-1.71)	.02
Education level at or above secondary school graduation	197 (78.6)	.19			314 (64.7)	<.001	0.54 (0.44-0.67)	<.001	1039 (78.5)	<.001	1.44 (1.20-1.74)	<.001	281 (74.6)	.80		
Religion very or fairly important	78 (31.2)	.50			151 (31.1)	.34			341 (25.8)	<.001	0.68 (0.57-0.82)	<.001	145 (38.5)	<.001	1.62 (1.29-2.04)	<.001
Current smoker	53 (21.2)	.18			86 (17.7)	.81			239 (18.1)	.94			63 (16.7)	.45		
Time of inclusion, mean (SD), mo	10.09 (5.86)	<.001	0.99 (0.99-0.99)	<.001	12.89 (5.91)	.18			12.84 (5.95)	.01	1.01 (1.00-1.01)	.009	12.80 (6.02)	.41		

Abbreviations: aOR, adjusted odds ratio; DS, down syndrome; IT, invasive testing; NIPT, noninvasive prenatal testing.

^a^ Cluster 1 was characterized by participants who wanted all possible information as soon as possible (250 women [10.3%]); cluster 2, participants who wanted all possibilities of getting information (485 women [19.9%]); cluster 3, participants who were primarily concerned with information on DS (1324 women [54.3%]); and cluster 4, participants characterized by aversion to risk of fetal loss (377 women [15.5%]).

^b^ *P* values from univariate logistic regressions.

### Was Decision Making in Line With Attitudes?

Overall, pregnant women’s decisions were consistent with their attitudes (Table 4). Women who refused participation in the trial prior to randomization were primarily found to be included in cluster 1 (78.8% vs 11.3% distributed among the other clusters; *P* < .001). These women were mainly characterized with higher nuchal translucency measurements by ultrasonography (median at 0.80 vs 0.67; *P* = .003) and fewer experiences of fetal loss (13.2% of women in cluster 1 reporting 0 or 1 previous experience of fetal loss vs 9.9% in the other women; *P* = .001). As for refusal of randomized assignment to IT, the lowest rate was in cluster 1 (1.2%), while the highest was in cluster 4 (44.3%). In all, 43.2% of women in cluster 4, representing 6.7% of the surveyed women, declined to participate in the trial either before or after randomization. In addition, most women opting for NIPT after having declined IT were included in clusters 3 and 4 (60.4% and 22.6%, respectively, vs 17.0% for the other clusters; *P* < .001).

**Table 4.** Actual Decision-Making and Visual Analog Scale Values Attached to IT Depending on Clusters for 2436 Pregnant Women at High Risk for Fetal Down Syndrome^a^

Decision	Total, No. (%)	Cluster 1 (n = 250)		Cluster 2 (n = 485)		Cluster 3 (n = 1324)		Cluster 4 (n = 377)	
		No. (%)	*P* Value	No. (%)	*P* Value	No. (%)	*P* Value	No. (%)	*P* Value
Refused participation prior to randomization between IT and NIPT	445 (18.3)	197 (78.8)	<.001	52 (10.7)	<.001	146 (11.0)	<.001	50 (13.3)	.006
Refused their randomized assignment to IT	255 (10.5)	3 (1.2)	<.001	34 (7.0)	.005	105 (7.9)	<.001	113 (30.0)	<.001
Accepted their randomized assignment to:									
IT	750 (30.8)	26 (10.4)	<.001	176 (36.3)	.003	491 (37.1)	<.001	57 (15.1)	<.001
NIPT	979 (40.2)	22 (8.8)	<.001	223 (46.0)	.004	579 (43.7)	<.001	155 (41.1)	.69
Opted for NIPT after having declined IT^b^	506 (20.8)	5 (1.9)	.59	73 (15.1)	.67	799 (60.4)	<.001	85 (22.6)	<.001

Abbreviations: IT, invasive testing; NIPT, noninvasive prenatal testing.

^a^ Cluster 1 was characterized by participants who wanted all possible information as soon as possible (250 women [10.3%]); cluster 2, participants who wanted all possibilities of getting information (485 women [19.9%]); cluster 3, participants who were primarily concerned with information on DS (1324 women [54.3%]); and cluster 4, participants characterized by aversion to risk of fetal loss (377 women [15.5%]).

^b^ Calculations based on the 255 pregnant women who refused their randomized assignment to IT.

## Discussion

The present study confirmed pregnant women’s preference toward NIPT^18,19,20,21,22,23,24,25,26,27^ and showed highly contrasted attitudes toward IT and NIPT in pregnant women at high risk of fetal DS by combined first-trimester screening. A clear-cut preference for IT (cluster 1) was associated with risk aversion to IT-related fetal loss being outweighed by aversion to ambiguous information on fetal chromosomal abnormalities other than DS, leading to lower VAS scores for NIPT. In contrast, clear-cut preference for NIPT (cluster 4) was expressed by women with strong aversion to IT-related risk of fetal loss, confirming previous findings^18,19,24,25^ and being consistent with highest VAS scores attached to NIPT. In between, attitudes in clusters 2 and 3 involved both risk aversion and ambiguity aversion to different extents.

In addition, pregnant women’s trade-offs between pregnancy risk taking and extent of information seeking were found to differ depending on DS risk levels. Women who considered their own risk of fetal DS following combined test results as being rather low were more likely to report strong aversion to IT-related risk (cluster 4). In contrast, those with higher nuchal translucency measurements were more likely to report strong aversion to ambiguity conveyed by NIPT results (cluster 1). While DS risk assessment is combining multiple parameters (age, nuchal translucency, and biochemical markers), a common behavioral bias, the base rate fallacy bias,^28^ could explain attitudes in cluster 1. Base rate fallacy bias occurs when focusing on specific information, ie, the ultrasonography component of DS risk, rather than on overall information on DS risk and making inferences about the outcome. As a result, ultrasonographic screening could shape high-risk women’s attitudes, just as age has been shown to shape attitudes toward IT among low-risk pregnant women.^29^ Otherwise, the present study mainly confirmed the role played by education and religious beliefs on attitudes.^25,30^

Relevance of risk aversion and ambiguity aversion in explaining attitudes was highlighted by high consistency of attitudes with decision-making. Women with clear-cut preference for IT (cluster 1) were also more likely to decline to participate in the clinical study, suggesting that these women acknowledged the uncertain component of the information provided by NIPT and expressed strong aversion to ambiguity generated by incomplete information from NIPT. Another main finding related to the fact that few women underwent IT despite negative NIPT results, although approximately 70% of women considered undergoing IT in addition to NIPT as their preferred option. While undergoing IT in addition to NIPT did not involve any monetary constraints, the possibility of base rate fallacy bias cannot be ruled out. Indeed, negative NIPT results could lead pregnant women to implicitly extrapolate on the other chromosomal abnormalities not targeted by NIPT. In view of the false reassurance possibly induced by negative NIPT results, further investigations should be conducted on the extent to which base rate fallacy bias truly affects pregnant women’s decisions. Otherwise, this study shows that the availability of NIPT may reduce the refusal rates for further testing,^31^ far from the commonly observed 20% rate of high-risk pregnant women’s refusal of IT when NIPT was not available.^32,33,34^ Lastly, this study also shows that approximately a third of pregnant women anticipated their decision in the case of high risk of DS^21^ and that older pregnant women attached higher value to NIPT than their younger counterparts.^23^

### Limitations

Attitudes and behaviors were studied within the framework of a clinical trial, in which counseling is likely to have been homogeneous and consistent in informing pregnant women about their options.^35^ Informed decisions were therefore likely to have been facilitated, possibly beyond what would be seen in routine antenatal practice. Another limitation was that DS was the only chromosomal abnormality targeted by NIPT, although trade-offs between pregnancy risk taking and extent of information seeking are likely to stand unless NIPT targets all chromosomal abnormalities detectable using IT. Another limitation relates to the rapidly evolving field of molecular cytogenetics that has recently identified comparative genomic hybridization microarray to provide additional information to the standard karyotype following IT. Although complete information should include genomic hybridization microarray on trophoblast or amniotic fluid, this option was not considered in our study. A final limitation concerned the risk of miscarriage of 0.5% to 1.0% associated with IT that was included in the information provided. There is now strong evidence that this risk is likely to be overstated.^14,36,37^

## Conclusions

The present study highlights the major role played by risk aversion and ambiguity aversion in shaping attitudes and decision making. Availability of NIPT imposes complex decisions and informed decision making that would require receiving extensive information on abnormalities targeted by both NIPT and IT. Genetic counseling goes beyond the trade-off between first-line combined screening and invasive procedure-related risk. While pregnant women have been shown to greatly differ in terms of extent of information seeking, tolerance for uncertainty, and pregnancy risk taking, counseling should involve listening to women’s preferences as to what they want to get out of prenatal testing.

## Notes

Supplement. **eAppendix.** Questionnaire
