# Supplementary material for: Women’s Attitudes Toward Invasive and Noninvasive Testing When Facing a High Risk of Fetal Down Syndrome
Source: JAMA Netw Open. 2019 Mar 29;2(3):e191062. doi: 10.1001/jamanetworkopen.2019.1062 (PMC6450316; doi:10.1001/jamanetworkopen.2019.1062)
Supplement: Supplement. — eAppendix. Questionnaire [file jamanetwopen-2-e191062-s001.pdf]

Supplementary Online Content

Seror V, L’Haridon O, Bussières L, et al; SAFE 21 Study Group. Women’s attitudes toward invasive and noninvasive testing when facing a high risk of fetal Down syndrome. *JAMA Netw Open*. 2019;2(3):e191062. doi:10.1001/jamanetworkopen.2019.1062

eAppendix. Questionnaire

This supplementary material has been provided by the authors to give readers additional information about their work.

© 2019 Seror V et al. *JAMA Network Open.*

SAFE 21 STUDY

PATIENT'S CODE

PATIENT'S LABEL

We are currently conducting a survey of pregnant women who, just like you, have just had the result of trisomy 21 screening by ultrasound and blood test. We understand that this information can be very disturbing. We hope that this questionnaire will help you make the decisions that suit you best..

You will see that there are some questions about the screening tests you have just done and several other questions about your attitudes towards the options that are available to you. Completing this questionnaire should take you about 10 minutes..

Whatever decision you decide to take, your opinion is very important so that the medical follow-up of the pregnancies is done in the best conditions and that it takes into account the point of view of the women.

Your answers are strictly confidential and will not be shared with the medical team. This questionnaire is not a substitute for medical consultation and you can ask any questions you want to the doctor or midwife. At any time, you remain free and autonomous to perform or not the tests that can be offered.

Your answers will be analysed anonymously. The grouped responses of all participants in the study will be the only results available. It will then be impossible to identify the participants, in accordance with the provisions of the Data Protection Act. You can amend or cancel your answers for the duration of the study.

© 2019 Seror V et al. *JAMA Network Open.*

FIRST TRIMESTER ULTRASOUND EXAMINATION

Ultrasound examination in the 1st trimester of pregnancy is a touching moment for pregnant women (couples), but it is also a medical act that makes it possible to check the age of pregnancy but also to make a first screening for serious malformations and to assess the risk of fetal trisomy 21.

1. Who was looking after your pregnancy by the time you did the 1st trimester ultrasound?

☐ A general practioner
☐ A gynecologist or an obstetrician in the private sector
☐ An obstetrician within a maternity unit
☐ A midwife within a maternity unit

2. Did you decide to do the 1st trimester ultrasound examination before the doctor (the midwife) suggested it?

☐ Yes
☐ No, I did not think about it until the doctor (the midwife) suggested it
☐ No, I did not know we could do an ultrasound examination in the first trimester of pregnancy

3. Did the doctor or midwife explain to what this ultrasound examination was about ?

☐ Extensive explanations
☐ Few explanations
☐ No explanation

4. Would you have liked more explanations ?

☐ YES
☐ NO

5. How did the doctor (the midwife) advise you about the ultrasound examination ?

☐ He (she) told me what to do
☐ He (she) gave me his opinion and told me to decide
☐ He (she) told me that it was up to me to decide and that he (she) did not have to give his opinion

6. How did you decide to do the ultrasound?

☐ I let the doctor (the midwife) decide because he (she) knows better than me what to do
☐ I decided with the help of the doctor (the midwife)
☐ I decided without the help of the doctor (the midwife)

7. Did the doctor's (midwife's) explanations help you make your decision?

☐ YES
☐ NO

8. Was it easy or difficult for you to decide to do the ultrasound examination ?

☐ It was an easy decision
☐ It was a difficult decision
☐ I did not feel like I was making a decision

© 2019 Seror V et al. *JAMA Network Open.*

DETECTION OF TRISOMY 21 BY BLOOD TEST

To find out the risk of trisomy 21 more precisely, a blood test has been proposed to you as to all pregnant women, in addition to ultrasound.

9. Who was looking after your pregnancy at the time you were tested for Trisomy 21 by blood test?

☐ The same doctor (midwife) as for the 1st trimester ultrasound examination.
☐ A general practioner
☐ A gynecologist or an obstetrician in the private sector
☐ An obstetrician within a maternity unit
☐ A midwife within a maternity unit

10. Did you decide to do the test before the doctor (the midwife) suggested it?

☐ YES
☐ No, I did not think about it until the doctor (the midwife) suggested it ⍰
☐ I did not know that trisomy 21 could be tested by a blood test

11. Did the doctor or midwife give you an explanation about the test?

☐ Extensive explanations
☐ Few explanations
☐ No explanation

12. Would you have liked more explanations ?

☐ YES
☐ NO

13. How did the doctor (the midwife) advise you about the ultrasound examination ?

☐ He (she) told me what to do
☐ He (she) gave me his opinion and told me to decide
☐ He (she) told me that it was up to me to decide and that he (she) did not have to give his opinion

14. How did you decide to do the ultrasound?

☐ I let the doctor (the midwife) decide because he (she) knows better than me what to do
☐ I decided with the help of the doctor (the midwife)
☐ I decided without the help of the doctor (the midwife)

15. Did the doctor's (midwife's) explanations help you make your decision?

☐ YES
☐ NO

16. Was it easy or difficult for you to decide to do the ultrasound examination ?

☐ It was an easy decision
☐ It was a difficult decision
☐ I did not feel like I was making a decision

© 2019 Seror V et al. *JAMA Network Open.*

RESULTS OF DOWN SYNDROME SCREENING

The result of trisomy 21 screening takes into account both the ultrasound, the blood test and your age. Pregnant women are often anxious when the screening result is announced. The following questions may help you identify points for which you would like more explanations from your doctor (midwife).

17. Did the doctor or midwife give you an explanation about the result of Down Syndrome screening ?

- ☐ Extensive explanations
- ☐ Few explanations
- ☐ No explanation

18. Est- Would you have liked more explanations ?

- ☐ YES
- ☐ NO

19. What do you think screening shows?

- ☐ I understood that the risk of trisomy 21 is rather low
- ☐ I understood that the risk of trisomy 21 is high
- ☐ I understood that the risk of trisomy 21 is very high
- ☐ Other:

20. While waiting for the trisomy 21 screening result by the BLOOD TEST, did you think back to your decision according to the result?

- ☐ YES
- ☐ NO

f you answered "YES" to question 20, what did you decide?

- ☐ I decided to wait for the result to make my decision
- ☐ I decided to do a diagnostic examination (karyotype by trophoblast biopsy or amniocentesis) regardless of the result
- ☐ I decided not to do a diagnostic examination (karyotype by trophoblast biopsy or amniocentesis) whatever the result
- ☐ I decided to do what the doctor (the midwife) would tell me to do

© 2019 Seror V et al. *JAMA Network Open.*

YOUR ATTITUDE TOWARDS THE DIAGNOSTIC EXAMINATION

The diagnostic test makes it possible to know with CERTAINTY if there is a trisomy 21 or another anomaly of the chromosomes .
To do the test, a sample of the young placenta (trophoblast biopsy) or amniotic fluid (amniocentesis) has to be obtained by introducing a needle into the uterus. Because of this procedure, there is a risk of miscarriage that occurs in about 1 in every 100 women.
The following questions may help you make the decisions that suit you best.

21. In your opinion, is a miscarriage following the diagnostic examination the worst that can happen?

- ☐ YES
- ☐ No, it is the birth of a child with Down syndrome 21 that is the worst

22. What do you think of the following proposals?

"Because of the risk of miscarriage, one should not perfom the diagnostic examination"

o Strongly agree
o Mostly agree
o Mostly disagree
o Not at all

"It is better to take the risk of having a miscarriage than to be worried until the end of pregnancy"”

o Strongly agree
o Mostly agree
o Mostly disagree
o Not at all

"From the moment the blood test was made, the diagnostic examination must be done if the doctor (the midwife) proposes it”

o Strongly agree
o Mostly agree
o Mostly disagree
o Not at all

“Il ne faut pas faire l'examen de diagnostic si on a décidé de ne pas faire d'interruption de grossesse en cas de trisomie 21”

o Tout à fait d'accord
o Plutôt d'accord
o Plutôt pas d’accord
o Pas d’accord du tout

"Do not do the diagnostic examination if you decide not to terminate your pregnancy in case of trisomy 21"

o Strongly agree
o Mostly agree
o Mostly disagree
o Not at all

© 2019 Seror V et al. *JAMA Network Open.*

YOUR ATTITUDE TOWARDS THE NEW DETECTION TEST FOR TRISOMY 21

A new test by blood test could avoid the diagnostic examination, and therefore the risk of miscarriage (in about 1 in 100 women). This new test allows to have information ALMOST CERTAIN on trisomy 21: it identifies 99% of trisomies 21. The diagnostic examination is only proposed when trisomy 21 is identified.

This new test does not make it possible to know if there are other chromosomal anomalies that the trisomy 21. To know it, it is necessary to perform the diagnostic examination. These other abnormalities are very infrequent but some of them may require specific medical management.

23. What do you think is the worst?

- ☐ Take a risk of miscarriage that occurs in about 1 in 100 women ▯
- ☐ No information on chromosomal abnormalities other than trisomy 21 in about 1 in 100 women

24. What do you think is best for you?

- ☐ Take a risk of miscarriage (in about 1 in every 100 women) and have some information on ALL chromosomal abnormalities ▯
- ☐ DO NOT take any risks and have information ALMOST CERTAIN about trisomy 21 ONLY

25. Imagine that you have a choice of the following three options. Which one do you prefer?

- ☐ Option 1: Refuse to do neither the new test nor the diagnostic test
- ☐ Option 2: Do the new test
- ☐ Option 3: Do the diagnostic examination

© 2019 Seror V et al. *JAMA Network Open.*

YOUR ATTITUDE TOWARDS THE SCIENTIFIC STUDY ON THE NEW TEST

The doctor (the midwife) suggests that you participate in a scientific study that gives you the opportunity to perform the new test. This new test gives information ALMOST CERTAIN about the presence or the absence of trisomy 21.

All pregnant women participating in the study will continue to be seen by the same doctors (midwives). Refusing to participate in the scientific study has no negative consequence: the monitoring of the pregnancy will have the same quality and will be done by the same doctors (midwives).

26. **What is your attitude towards this scienti fi c study?**

Tick the box that best suits you.
We remind you that your answers will not be sent to the medical team.

- ☐ I do not want to do the diagnostic test nor the new test
- ☐ I would like to do the new test, but if it is not possible, I would refuse the diagnostic test
- ☐ I would like to do the new test, but I also want to be able to do the diagnostic test
- ☐ I would like to do the new test, but I am afraid that I will be told afterwards that it is not possible to do the diagnostic test
- ☐ I do not want this new test: I want the diagnostic test

27. **If you had only the choice between the new test and the diagnostic test, what would be your preference on a scale of 1 to 10?**

I definitely prefer the new test	I do not have any preference	I definitely prefer the diagnostic test

0 1 2 3 4 5 6 7 8 9 10

© 2019 Seror V et al. *JAMA Network Open.*

TO CONCLUDE …

28. How old are you ?

I am ……….

29. Do you have children ?

- ☐ YES **if YES, how many ?** I have……. Child/Children
- ☐ NO

30. Have you done the diagnostic examination (trophoblast biopsy or amniocentesis) for a previous pregnancy?

- ☐ No, this is my first pregnancy
- ☐ No, the doctor did not suggest it
- ☐ No, because I did not have trisomy 21 screening (neither ultrasound nor blood test)
- ☐ Yes, because the doctor had suggested it
- ☐ Yes, because I asked for it
- ☐ Other

31. Is religion important to you?

- ☐ Very important
- ☐ Somewhat important
- ☐ Not important
- ☐ Not important at all

32. What is the highest degree you have obtained ?

- ☐ No diploma
- ☐ Certificate of Studies / Certificate of Primary Studies / CEP
- ☐ BEPC / College Certificate / Undergraduate Certificate
- ☐ BEP / CAP
- ☐ Bachelor
- ☐ Bac + 1 (1st year of license, L1, 1st year of DEUG, IUT, BTS, preparation ...)
- ☐ Bac +2 (2nd year of license, L2, 2nd year of DEUG, IUT, BTS, preparation ...)
- ☐ Bac +3 (3rd year of license, L3, ex-license, high school ...)
- ☐ Bac + 4 (master 1, M1, master, high school ...)
- ☐ Bac +5 or more (master 2, M2, DESS, DEA, PhD, MBA ...)
- ☐ Other degree (specify): ....

33. Any comments

We thank you for answering this questionnaire

© 2019 Seror V et al. *JAMA Network Open.*
